# PMPred-AE: a computational model for the detection and interpretation of pathological myopia based on artificial intelligence

**DOI:** 10.3389/fmed.2025.1529335

**Published:** 2025-03-13

**Authors:** Hong-Qi Zhang, Muhammad Arif, Maha A. Thafar, Somayah Albaradei, Peiling Cai, Yang Zhang, Hua Tang, Hao Lin

**Affiliations:** ^1^The Clinical Hospital of Chengdu Brain Science Institute, School of Life Science and Technology, University of Electronic Science and Technology of China, Chengdu, China; ^2^College of Science and Engineering, Hamad Bin Khalifa University, Doha, Qatar; ^3^Computer Science Department, College of Computers and Information Technology, Taif University, Taif, Saudi Arabia; ^4^Department of Computer Science, Faculty of Computing and Information Technology, King Abdulaziz University, Jeddah, Saudi Arabia; ^5^School of Basic Medical Sciences, Chengdu University, Chengdu, China; ^6^Innovative Institute of Chinese Medicine and Pharmacy, Academy for Interdiscipline, Chengdu University of Traditional Chinese Medicine, Chengdu, China; ^7^School of Basic Medical Sciences, Southwest Medical University, Luzhou, China; ^8^Central Nervous System Drug Key Laboratory of Sichuan Province, Luzhou, China

**Keywords:** myopia, pathological myopia, deep learning, EfficientNetv2, Grad-CAM

## Abstract

**Introduction:**

Pathological myopia (PM) is a serious visual impairment that may lead to irreversible visual damage or even blindness. Timely diagnosis and effective management of PM are of great significance. Given the increasing number of myopia cases worldwide, there is an urgent need to develop an automated, accurate, and highly interpretable PM diagnostic technology.

**Methods:**

We proposed a computational model called PMPred-AE based on EfficientNetV2-L with attention mechanism optimization. In addition, Gradient-weighted class activation mapping (Grad-CAM) technology was used to provide an intuitive and visual interpretation for the model’s decision-making process.

**Results:**

The experimental results demonstrated that PMPred-AE achieved excellent performance in automatically detecting PM, with accuracies of 98.50, 98.25, and 97.25% in the training, validation, and test datasets, respectively. In addition, PMPred-AE can focus on specific areas of PM image when making detection decisions.

**Discussion:**

The developed PMPred-AE model is capable of reliably providing accurate PM detection. In addition, the Grad-CAM technology was also used to provide an intuitive and visual interpretation for the decision-making process of the model. This approach provides healthcare professionals with an effective tool for interpretable AI decision-making process.

## 1 Introduction

Pathological myopia (PM) is a serious visual disease that can lead to irreversible visual damage or even blindness (1–3). In recent years, PM has become one of the main causes of visual impairment and permanent blindness worldwide, especially in Asian countries. According to the research by Holden et al. (4), by 2050, nearly half of the global population will be affected by myopia, with approximately 10% suffering from high myopia, which will also become the leading cause of permanent blindness. In addition, retinopathy and complications related to myopia may also increase the risk of visual damage (5–7). Therefore, timely diagnosis and early detection of PM are crucial. Currently, develop an automated, accurate, and non-invasive method PM diagnosis method is an urgent task.

With the development of artificial intelligence (AI) and the accumulation of myopia data, a variety of computational methods have been developed (8–10). For example, Liu et al. (10) introduced a method using texture features and Support Vector Machine (SVM) (11–13) to automatically detect PM. This method processed retinal fundus images by extracting region of interest (ROI) and detecting the optic nerve head. Subsequently, texture-based metrics were generated, categorized and grouped into zones for context-based generation of features. Finally, SVM was used to detect PM based on these features, achieving an accuracy (ACC) of 87.5% (14). Zhang et al. (15) proposed an automatic detection method for PM based on max-relevance and min-redundancy (mRMR). This method built a feature space from information extracted from fundus images and medical screening data, created a ranked feature library using mRMR, searched for the most compact feature set with a forward selection wrapper, and then used SVM for detection. As a result, they achieved an ACC of 89.3% for the right eye and 88.5% for the left eye (15). Xu et al. (16) developed a detection method for PM based on bag-of-feature and sparse learning. During the training phase, the codebook for the bag-of-feature model and the classification model were learned, and the top related visual features were discovered through sparse learning.

In the detection phase, local features were first extracted from a given retinal fundus image, quantified using the learned codebook to obtain global features. Finally, the classification model was used to determine the presence of PM, achieving an ACC of 90.6% (16). Zhang et al. (17) also developed an automatic diagnostic method for PM based on heterogeneous biomedical data, integrating data from various sources including imaging data, demographic/clinical data, and genotyping data, and ultimately using a multiple kernel learning (MKL) approach to accurately detect PM, achieving an average Area Under Curve (AUC) of 0.888. Chen et al. (18) introduced a deep learning architecture for automating the diagnosis of glaucoma. This method used a convolutional neural networks (CNN) (19, 20) model with four convolutional layers and two fully connected layers, combined with dropout and data augmentation strategies to enhance diagnostic performance. The method achieved AUC values of 0.831 and 0.887 on the ORIGA and SCES datasets, respectively (18). Xu et al. (21) proposed an automated detection method for tessellated fundus based on texture features, color features and SVM. The method could achieve an ACC of 98%. Xu et al. (22) proposed a method for detecting ocular disease based on multiple informatics domains. This method combined pre-learned SVM classifiers effectively merging personal demographic data, genome information, and visual information from retinal fundus images. The final model obtained an AUCs of 0.935 for glaucoma, 0.822 for age-related macular degeneration (AMD), and 0.946 for PM (22). Septiarini et al. (23) introduced a method based on statistical features to automatically detect peripapillary atrophy in retinal fundus images. This method involved four steps: optic nerve head (ONH) localization, ONH segmentation, preprocessing, and features extraction. Through these steps, three key features were extracted: standard deviation (*σ*), smoothness (S), and third moment (μ3). By using a backpropagation neural network (BPNN), they achieved an ACC of 95% (23). Rauf et al. (24) proposed a CNN-based method for PM detection and obtained an ACC of 95%. Although these studies have achieved positive results, there are still several challenges: (1) Many advanced deep learning methods are emerging, but in the field of PM detection, these advanced technologies have not yet been applied. (2) Due to the uniqueness of the medical industry and the high requirements for model accuracy, model performance still needs to be improved. (3) Due to the differences in actual medical facilities, the efficiency of these models in poorly equipment medical environments is an important problem that needs to be overcome. (4) As an auxiliary diagnosis method, the interpretability of models was an important task, but current research in this area is still insufficient (25–28).

To address the aforementioned challenges, this study designed an improved model named PMPred-AE based on EfficientNetV2-L to automatically identify and diagnose PM. This study further enhanced the model’s ability to identify key features in the retina images by introducing the attention mechanism, thereby improving the accuracy of the diagnosis of PM. In order to provide visual explanations for the decision-making process of the model, we also adopted the Gradient-weighted class activation mapping (Grad-CAM) technique. Our study provides an efficient, accurate, and explainable model for the detection of PM.

## 2 Materials and methods

### 2.1 Dataset construction

The study utilized the PALM Challenge dataset, comprising training images, verification images and test images. The training dataset contains 187 non-PM and 213 PM. Similarity, the verification set consists of 400 images, with 189 labeled as non-PM and 211 as PM. Additionally, test set includes 400 images with corresponding labels: 187 categorized as non-PM and 213 as PM (29). This dataset configuration enabled rigorous evaluation and validation of the proposed methodologies.

### 2.2 Model design

The PMPred-AE architecture consists of two core components: a feature extractor and a classifier. In the feature extraction stage, we chose EfficientNetV2-L, an advanced CNN model aimed at accelerating image processing and improving its performance. As an upgraded version of the EfficientNet series, EfficientNetV2-L underwent pre-trained on a massive ImageNet dataset that covers millions of images and thousands of categories. Through its scalable architecture, EfficientNetV2-L cleverly balances the network depth, width, and resolution to achieve optimal performance and efficiency. EfficientNetV2-L is an upgraded version of the EfficientNet series. It optimizes the balance of network depth, width, and resolution to achieve high efficiency and accuracy in image processing tasks. Compared to advanced vision transformer (ViT) series’ ViT-L/16, EfficientNetV2-L achieves higher accuracy. Meanwhile, the training speed could increase by 7 times (30). In particular, the model utilizes lightweight depthwise separable convolution techniques, significantly reducing computational burden and model size while maintaining efficient feature extraction capabilities. Therefore, in the context of PM-detection, EfficientNetV2-L could efficiently identify key features in images and provide accurate data input for classifiers, significantly improving the performance of the model. Moreover, its superior computing speed and efficiency made it very suitable for application in medical environments with rudimentary equipment, providing strong technical support for early diagnosis and treatment. In the classification stage, we used an improved fully-connected neural network based on the attention mechanism. The core function of this improvement is to enhance the model’s attention to the most important parts of the input features. By assigning different weights to the input features, the attention mechanism allows the model to prioritize the features that contribute the most to the final classification decision, rather than treating all input features equally. This dynamic weight allocation method not only improves the model’s understanding of the data, but also increases the adaptability and flexibility of the model, enabling it to automatically focus on the most critical information. Specifically, we used a linear layer to transform all the features into a one-dimensional space, and then map them to a value between 0 and 1 using the Softmax function. Finally, this weight is multiplied by the original input features to emphasize the features that contribute the most to the classification result. This improvement was particularly important for the detection of PM. It allows the model to pay special attention to the areas that revealed the pathological features of myopia. Through this mechanism, our model provided an efficient tool for the early diagnosis and treatment of PM.

### 2.3 Grad-CAM

In order to visually explain the decision-making process of CNN in PM detection tasks, we used Grad-CAM technique to generate a heatmap. Through Grad-CAM, we can clearly see which areas are given more attention when the model makes detection. This approach relies on the gradient information of the model, particularly focusing on the gradients of the feature layers from the last convolutional layer, to highlight the regions that contribute most to the model predictions. The working principle of Grad-CAM can be briefly described by the following mathematical expression.

First, for each channel in the feature layer A , the global average pooling of these slopes is calculated to obtain the weight coefficient (Equation 1):


(1) $$\alpha_{k}^{c}=\frac{1}{Z}\underset{i}{\sum }\underset{j}{\sum }\frac{\partial y^{c}}{\partial A_{ij}^{k}}$$

where, $y^{c}$ is the output score of the model for category c , $A_{ij}$ is the activation value of the feature layer at position $\left(i,j\right)$ , *k* is the *k*-th channel in the feature layer A, and Z is the total number of units in the feature layer.

Then, the weight coefficient is multiplied by the activation value of the feature layer and then accumulated. The final heatmap is generated by filtering through the *ReLU* function (Equation 2):


(2) $$L_{\mathrm{Grad}-CAM}^{c}=\mathrm{ReLU}\left(\underset{k}{\sum }\alpha_{k}^{c}A^{k}\right)$$

This process ensures that only features that have positive impact on l prediction category *c* of the mode were visualized, thereby enhancing the clarity and interpretability of the model’s decision. By applying Grad-CAM to the PMPred-AE model, the heatmap clearly reveals that the model focuses on the location of key pathological changes in the retina image when identifying PM. The heatmap provided by Grad-CAM not only demonstrates the reason behind the model’s high performance, but also proves its focusing ability, which is crucial to improve the reliability and trust of the model in practical medical applications. Through this way, Grad-CAM provides healthcare professionals with an intuitive tool to better understand and explain the decision-making process of the PMPred-AE, especially in medical diagnosis and treatment planning.

### 2.4 Parameter setting

The learning rate is set to 0.0001, the batch size is 8, the number of epochs is 50, and the optimizer is AdamW.

### 2.5 Evaluation index

Several widely used evaluation indicators (31–37), including precision (Pre) (Equation 3), recall (Rec) (Equation 4), accuracy (ACC) (Equation 5), F1-score (F1) (Equation 6), and Matthew’s coefficient of association (MCC) (Equation 7), were utilized to evaluate model’s performance, defined as follows:


(3) $$Pre=\frac{TP}{TP+FP}$$

(4) $$Rec=\frac{TP}{TP+FN}$$

(5) $$ACC=\frac{TP+TN}{TP+FP+TN+FN}$$

(6) $$F1=\frac{2\mathrm{PreRec}}{Pre+Rec}$$

(7) $$MCC=\frac{TP\times TN-FP\times FN}{\sqrt{\left(TP+FP\right)\left(TN+FN\right)\left(TP+FN\right)\left(TN+FP\right)}}$$

where *TP*, *TN*, *FP*, and *FN* represented the true positive, true negative, false positive, and false negative of the sample, respectively. We also drew the receiver operating characteristic curve (ROC) and precise recall curve (PRC), and obtained the area under the curve (AUC, AUPRC) (27, 38–41).

## 3 Results

### 3.1 Overview of experiment

**Figure 1:**
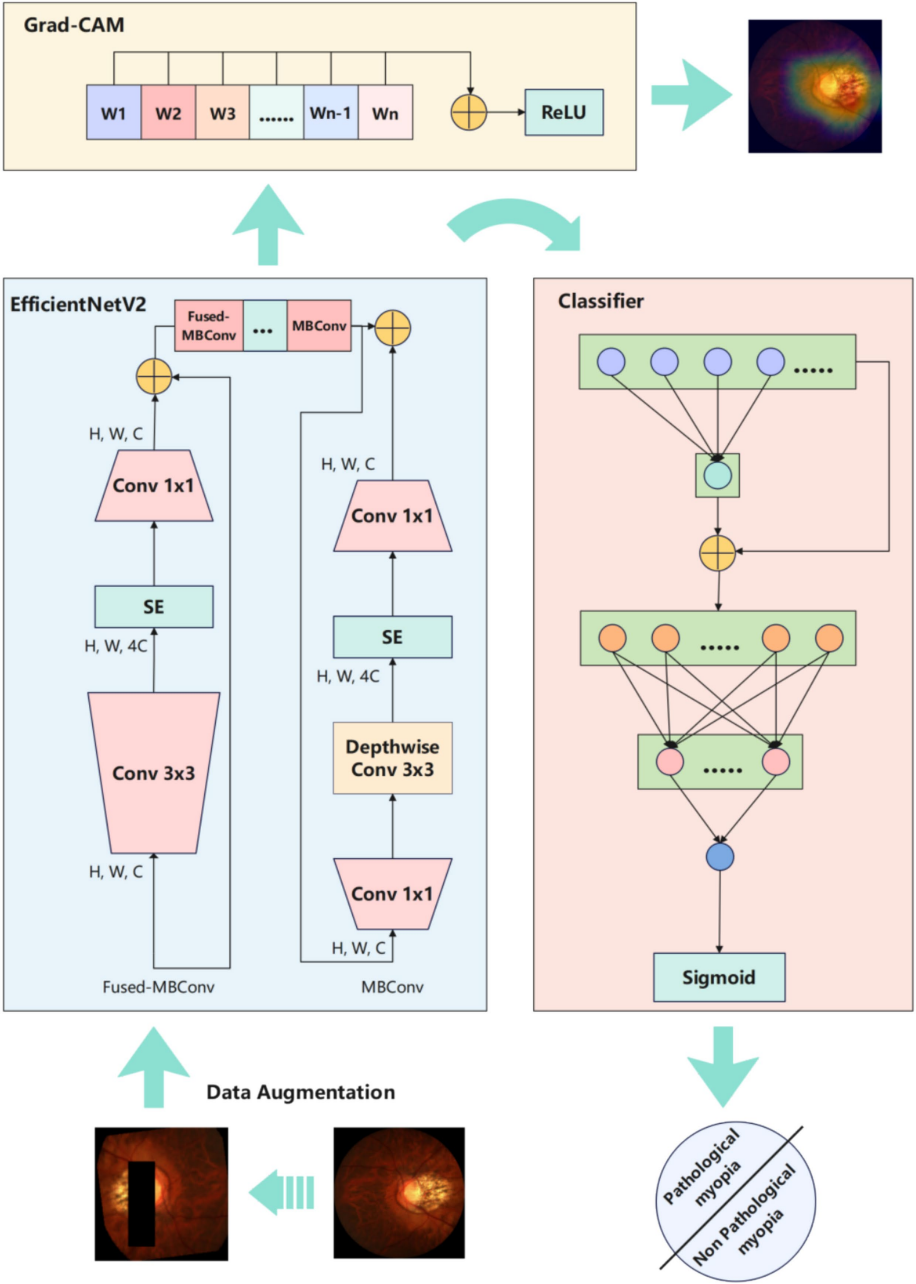
Experimental workflow overview diagram.

In our experiment, we first adopted data augmentation techniques to enrich and expand the original data set, and created more diverse training samples. Data enhancement included operations such as image rotation, resizing, and cropping. It was designed to simulate different shooting conditions and perspectives to improve the model’s generalization and robustness. The data-enhanced dataset was used to train our PMPred-AE model, which was based on the EfficientNetV2-L architecture and optimized to meet the specific requirements of PM-detection. EfficientNetV2-L is the foundation of our model. It has been pre-trained on the ImageNet data set, and therefore has strong feature extraction capabilities (42, 43). In order to further improve the performance of the model, we introduced an attention mechanism in the fully connected layer of the model. This mechanism enables the model to focus more on the key areas related to PM diagnosis in the image, thereby improving the accuracy of diagnosis. During the model training process, the model parameters were adjusted based on the performance on the verification set to achieve the optimal configuration. After training, we visualized the output of the model at different levels (shallow, middle, and deep). This step helped us understand how the model gradually extracted and utilized image features. In addition, we also used Grad-CAM technology to generate a heatmap that highlight the areas that the model focuses on when making predictions. In this way, we can not only verify the decision-making process of the model, but also provide intuitive visual explanations for doctors to help them better understand the basis of the model. Overall, our experiment combined data augmentation, attention mechanisms, and advanced model architecture and explanatory techniques to develop an efficient, accurate, and explainable model for the detection of PM (Figure 1).

### 3.2 Data augmentation

**Figure 2:**
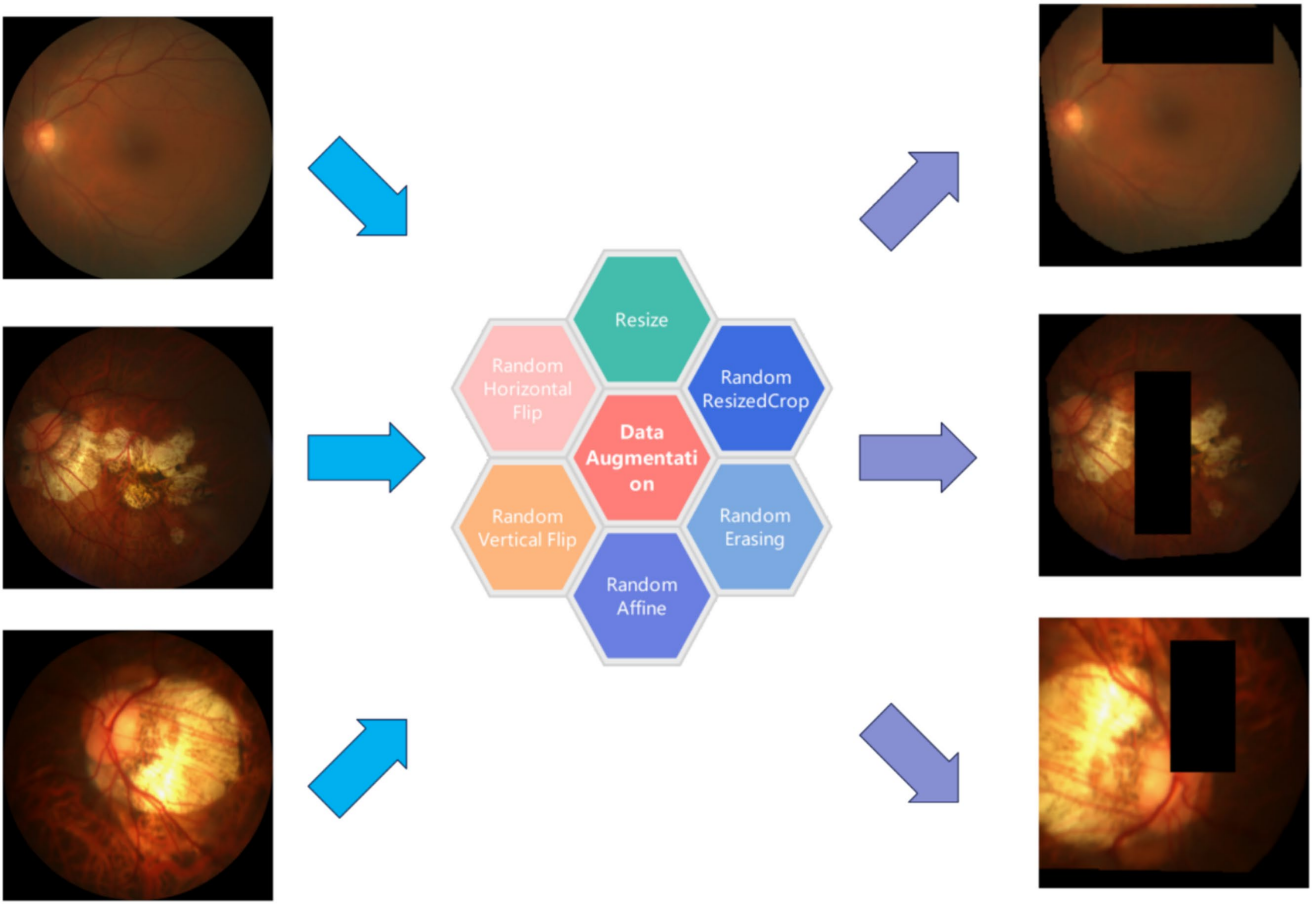
Data augmentation result diagram.

Due to the difficulty of collecting and annotating pathological images, only a small number of data samples could be collected under normal circumstances. Therefore, data augmentation was a very necessary task. It can effectively reduce the over-fitting degree of the model, and allow the model to learn more general knowledge instead of focusing too much on noise and some unique features, thereby improve the generalization and robustness of the model (44–46). In this study, we employed a combination approach for sample augmentation. The detailed procedure included initially resizing the images to 256×256 pixels. Subsequently, they are randomly cropped to 224×224 pixels. Then anti-aliasing techniques were applied to ensure image quality. In addition, to increase visual variety, the probability of horizontal and vertical flipping was set to 50%. This method also incorporated subtle random affine transformations, including rotations between −10 to 10 degrees, translations of 10% of the image width or height, and scaling between 90 to 110%. Furthermore, random erasure is applied with a 50% probability, randomly covering a small portion of the image, enhancing the model’s ability to handle image occlusion (Figure 2). Finally, the images were converted into tensors and normalized according to a specific mean and standard deviation to suit the needs of model training. We mainly used these methods to address the following issues: by randomly cropping and resizing, we simulated the scene where doctors observe the eyes from different distances and angles, and random rotation and affine transformation helped the model identify pathological features from multiple angles. Random erasure simulates potential occlusions during actual medical image acquisition. Normalization ensures consistency of image data during training, while anti-aliasing maintains the clarity of image details, which is crucial for identifying pathological features. By introducing various visual perturbations, this comprehensive data augmentation strategy facilitates the model in extracting valuable features from diverse image transformations, thereby enhancing performance and robustness in real-world application scenarios.

### 3.3 Model validation

**Figure 3:**
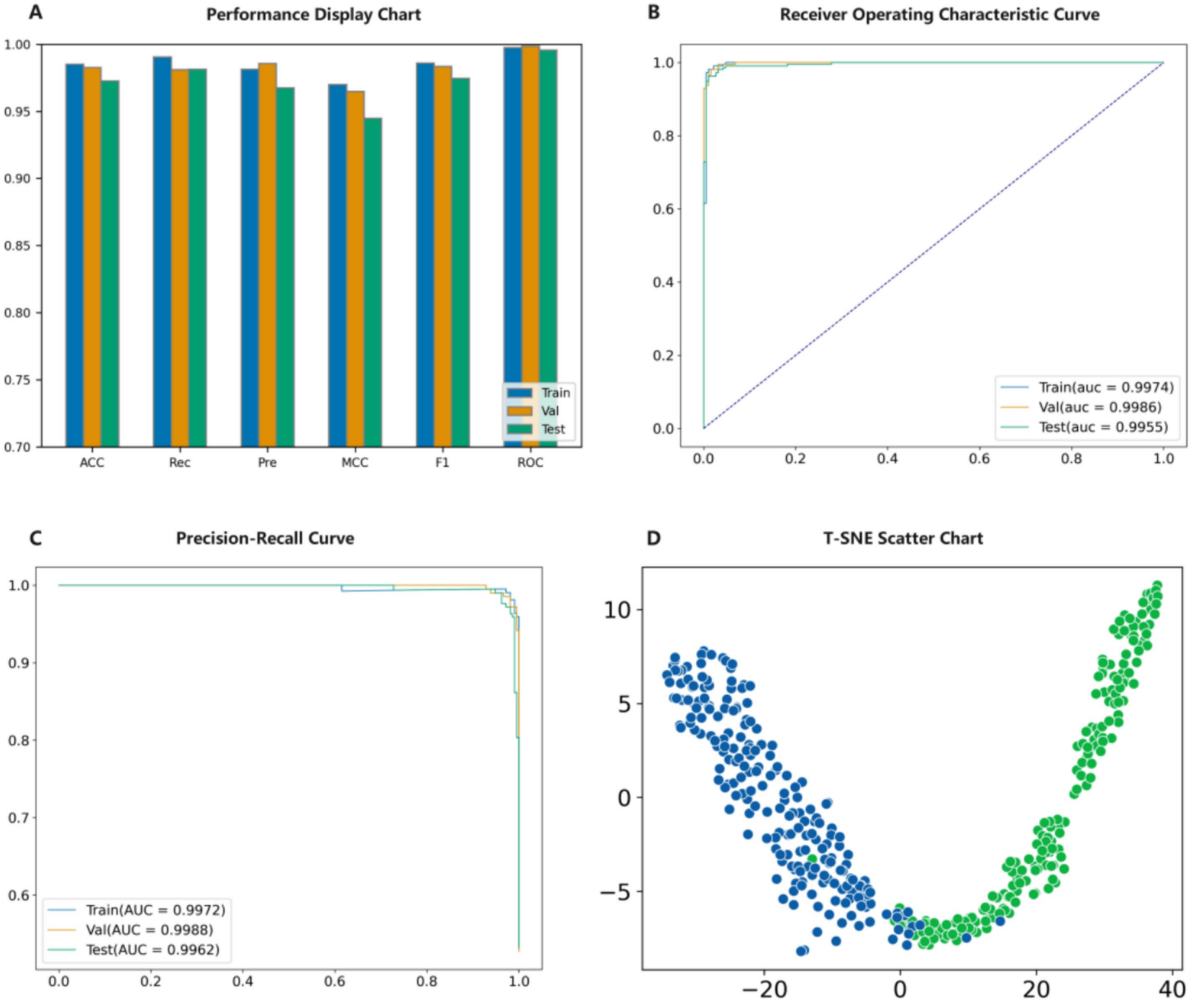
Model validation result diagram. **(A)** Evaluation results of the model. **(B)** ROC results of the model. **(C)** PRC results of the model. **(D)** t-SNE visualization the model.

A series of experiments have shown that the PMPred-AE model exhibits excellent performance in PM classification tasks. Firstly, the model is trained on the training set to ensure that it has sufficient learning foundation and can capture the key features and patterns in the data (47–50). Then, the validation set was used to adjust the parameters of the model, which further improved its performance and ensured its generalization ability on unseen data (51, 52). The experimental results showed that PMPred-AE performed well on the test set, and all evaluation indicators reached a very high level, such as ACC, F1, Pre, Rec and MCC with values of 0.9725, 0.9744, 0.9676, 0.9812 and 0.9448, respectively. This indicates that PMPred-AE has excellent ability to effectively distinguish PM from non-PM (Figure 3A, Table 1). In addition, by plotting ROC and PRC, we observed that the PMPred-AE model had good AUC and AUPRC under both conditions, with values of 0.9955 and 0.9962, respectively. This further demonstrated the efficiency of PMPred-AE model in feature extraction and capability in recognizing PM (Figures 3B,C). Finally, we used t-SNE technology to visualize the output of the model (Figure 3D) (53). The results showed that PM and non-PM can be clearly distinguished in a low-dimensional space, indicating that the model can effectively represent their features in a low-dimensional space and capture the complex patterns and structural differences between them. This further suggests that the PMPred-AE model has broad application prospect in clinical practice.

**Table 1:** The performance evaluation of model.

Method	ACC	Pre	Rec	F1	ROC	MCC
Train	0.9850	0.9814	0.9906	0.9860	0.9974	0.9699
Val	0.9825	0.9857	0.9810	0.9834	0.9986	0.9649
Test	0.9725	0.9676	0.9812	0.9744	0.9955	0.9448

### 3.4 Model explanations

**Figure 4:**
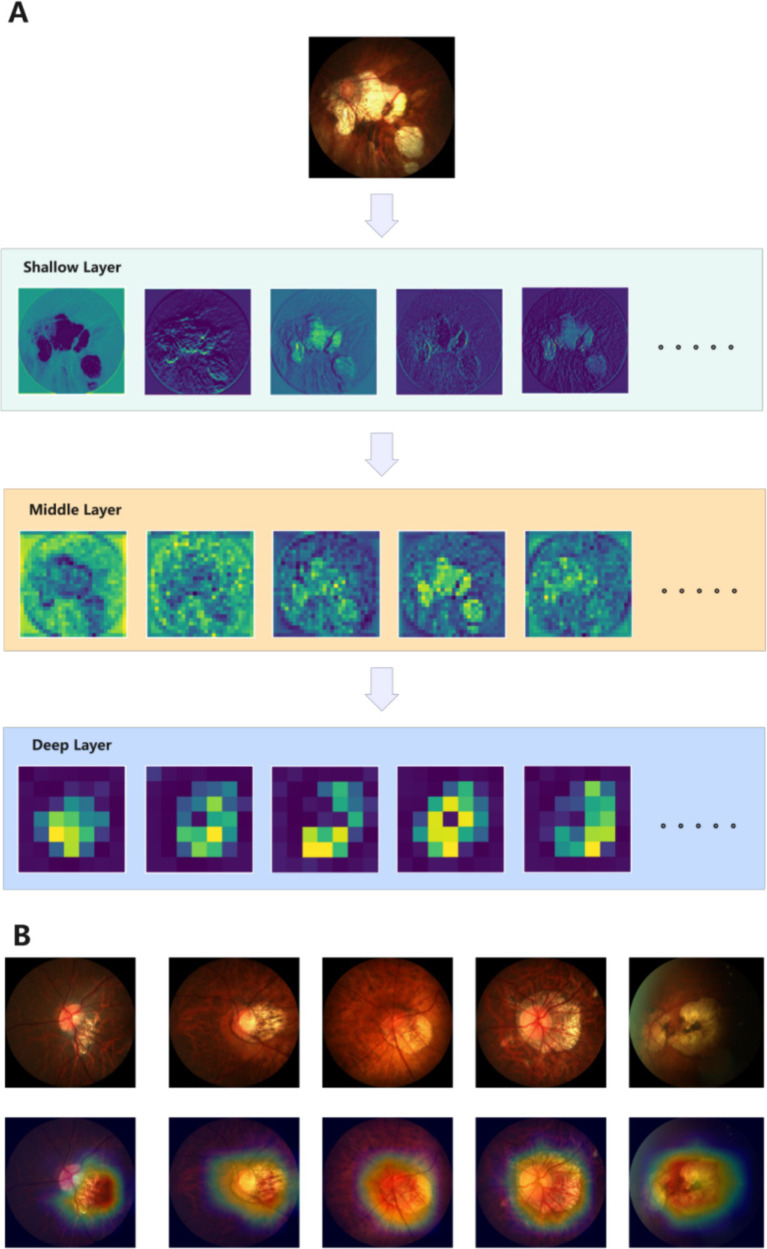
Model explanation display diagram. **(A)** Visualize the output results of shallow, middle, and deep layers of the model. **(B)** Visualization results of Grad-CAM.

To further confirm that PMPred-AE could effectively extract features, we visualized the output of the model’s shallow, middle, and deep layers. It can be clearly observed that as the depth of the model increases, the model can extract more abstract and higher-level features. This proves that the hierarchical structure of PMPred-AE model effectively promoted the gradual extraction and refinement of features (Figure 4A). Later, in order to further investigate why PMPred-AE could efficiently distinguish PM and non-PM, we used the Grad-CAM technology to generate a heatmap that could reveal the areas that the model focused on when making predictions, thus providing an explanation for the model’s decision-making process (Figure 4B). The heatmap revealed that the PMPred-AE model could effectively focus on the location of the key pathological changes in the image when identifying PM. These positions were often the key for distinguishing between PM and non-PM, which explained why the model could achieve high accuracy. This focusing ability not only improved the prediction performance of the model, but also increased its reliability and credibility in practical applications, especially in medical diagnosis and treatment planning.

### 3.5 Comparisons with existed works

**Figure 5:**
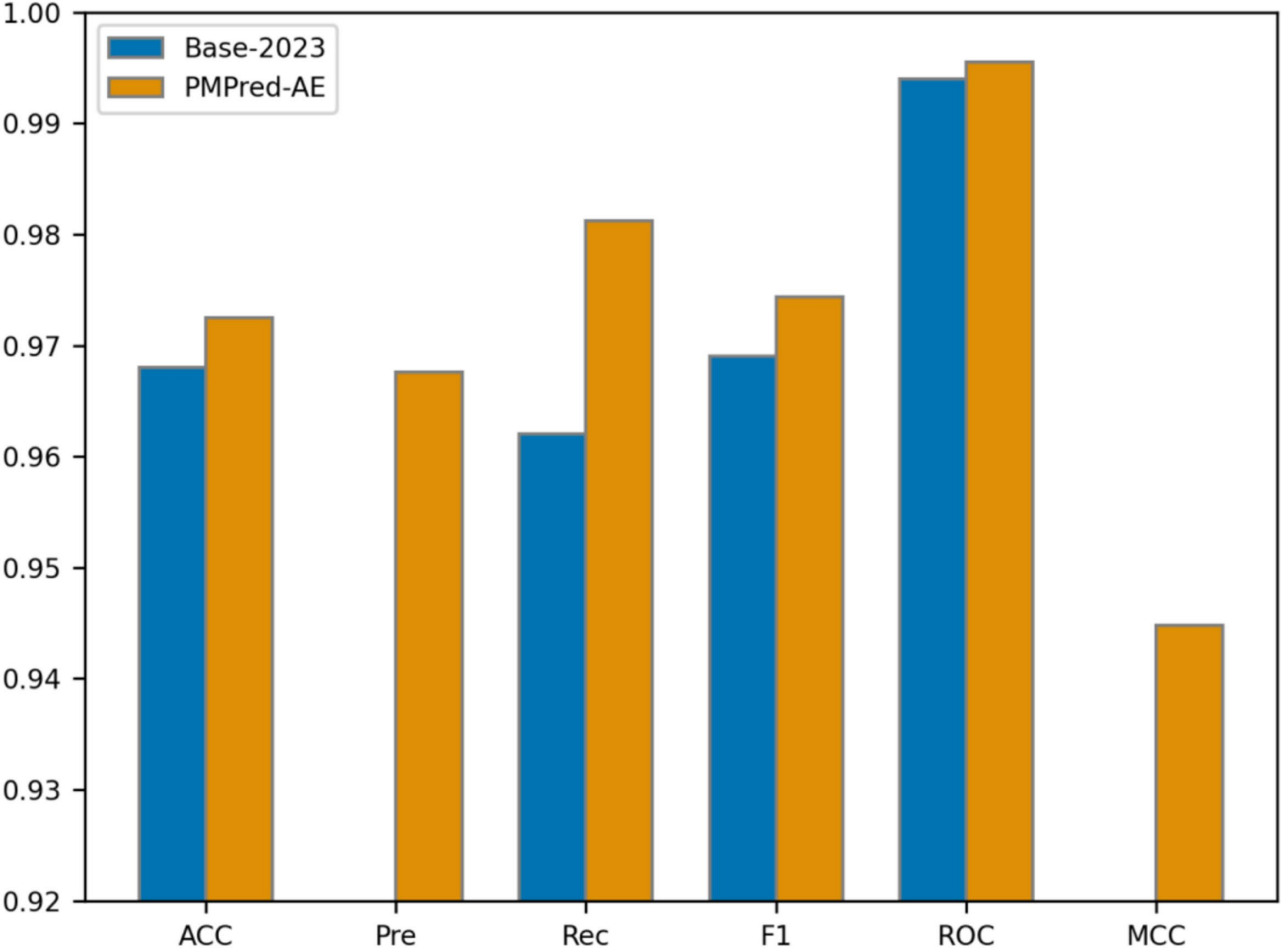
Comparison diagram of Base-2023.

To further demonstrate the performance of PMPred-AE in detecting PM, we should compare the proposed model with existed studies. However, those studies we mentioned earlier did not share their source code and used different datasets, making it impossible for use to make a fair comparison. Fortunately, we could use the PALM’s benchmark data from 2023 (Base-2023) (29). The experiment results showed that among all evaluation metrics, PMPred-AE is superior to Base-2023 (Figure 5, Table 2). By comparing with Base-2023, we further consolidated the validation of the PMPred-AE model and provided more reliable support for its application in clinical practice.

**Table 2:** Comparison with published results.

Method	ACC	Pre	Rec	F1	ROC	MCC
Base-2023	0.968	/	0.962	0.969	0.994	/
PMPred-AE	**0.9725**	**0.9676**	**0.9812**	**0.9744**	**0.9955**	**0.9448**

The bold font indicates the classifiers that work best.

## 4 Discussion

In this study, we designed an improved EfficientNetV2-L model based on the attention mechanism (PMPred-AE) for the automatic detection of PM. By using EfficientNetV2-L as the basic architecture for feature extraction and introducing improvements based on the attention-based mechanism in the classification stage, the PMPred-AE model could efficiently identify key features in eye image and significantly improve the prediction performance of the model. In the research, data augmentation techniques were used to expand the training samples, including image rotation, resizing, and cropping to improve the model’s generalization ability and reliability. In addition, Grad-CAM technology was introduced during the model training process to generate heatmaps, which provided a visual means to explain the decision process of the PMPred-AE in the identification of PM. The heatmap generated by Grad-CAM can clearly show the areas that the model focused on when making predictions, thereby enhancing the clarity and interpretability of the model’s decisions. Compared with existing work, PMPred-AE had a significant improvement in ACC, Rec, ROC, and F1. This confirmed its leading position in the field of PM-detection and provided strong support for its application in clinical practice.

The PMPred-AE model demonstrates significant potential and scalability in the field of medical image analysis. In addition to effectively detecting PM, PMPred-AE is also applicable to various medical imaging tasks, including the analysis of tumors, brain diseases, and lung diseases. Despite the unique characteristics of different medical images, PMPred-AE offers an efficient and interpretable framework that can be applied across diverse medical scenarios, showcasing substantial clinical application potential. The clinical value of PMPred-AE lies not only in its high accuracy and efficiency but also in its seamless integration with existing healthcare systems. The model can directly process images generated by standard medical devices without requiring additional workflows. Furthermore, PMPred-AE uses Grad-CAM technology to generate heatmaps that visualize the regions the model focuses on, helping physicians make more precise clinical decisions. The model’s lightweight design ensures efficient operation even in resource-constrained environments, making it particularly suitable for regions with limited healthcare resources. However, there are several challenges to be addressed in the deployment of PMPred-AE in practice. First, the quality and diversity of fundus images may vary due to differences in imaging devices and conditions, potentially affecting model performance. To address this, we can enhance the model’s generalization ability by expanding the training dataset and incorporating data augmentation techniques. Second, although the model employs an efficient network architecture, inference speed and computational resource requirements could become limiting factors in resource-constrained environments. To mitigate this, we plan to deploy the model on the cloud, leveraging cloud computing resources for inference to reduce the local computational burden. In summary, while the deployment of PMPred-AE faces several challenges, improvements in data quality, optimization of computational resources, and enhanced model robustness can effectively address these issues, ensuring the successful application of the model in clinical practice.

In summary, this research successfully developed an efficient, accurate, and explainable model for the detection of PM by combining advanced model architecture, attention mechanism, and explanatory techniques. This comprehensive method not only improved the performance of the model, but also provided a valuable reference for clinical diagnosis, demonstrating the great potential of deep learning in the field of medical image analysis. In the future, with the continuous advancement of algorithms and technology, such models are expected to play a greater role in improving the efficiency and accuracy of PM diagnosis. The source code has been uploaded to GitHub and can be accessed at: https://github.com/ZhangHongqi215/PMPred-AE.

## Data Availability

The original contributions presented in the study are included in the article/supplementary material, further inquiries can be directed to the corresponding authors.
